# Multiple Basal Cell Carcinomas in a Long-Term Survivor of Childhood ALL and HSCT—A Call for Dermatologic Vigilance

**DOI:** 10.3390/life16010055

**Published:** 2025-12-30

**Authors:** Elena Porumb-Andrese, Gabriela Stoleriu, Antonia Elena Huțanu, Cristian Mârţu, Mihaela-Paula Toader, Vlad Porumb, Cristina Colac-Boțoc, Ancuța Lupu, Gabriela Rusu-Zota, Emil Anton, Daciana Elena Brănișteanu

**Affiliations:** 1 Grigore T. Popa University of Medicine and Pharmacy, 16 Universitatii Str., 700115 Iasi, Romania; elena.andrese1@umfiasi.ro (E.P.-A.); martu.cristian@umfiasi.ro (C.M.); mihaela.toader@umfiasi.ro (M.-P.T.); vlad.porumb@umfiasi.ro (V.P.); cristina.botoc28@gmail.com (C.C.-B.); ancuta.ignat1@umfiasi.ro (A.L.); rusu.i.gabriela@umfiasi.ro (G.R.-Z.); emil.anton@umfiasi.ro (E.A.); daciana.branisteanu@umfiasi.ro (D.E.B.); 2 Faculty of Medicine and Pharmacy, “Dunarea de Jos” University of Galati, 14 Domneasca Str., 800008 Galati, Romania

**Keywords:** AI-assisted dermoscopy, basal cell carcinoma, childhood leukemia survivor, dermoscopy, graft-versus-host disease, hedgehog signaling, hematopoietic stem cell transplantation (HSCT), nicotinamide, secondary malignancies

## Abstract

(1) Background: Cutaneous secondary malignant neoplasms are a growing survivorship burden after pediatric cancers and hematopoietic stem cell transplantation (HSCT), yet skin-focused surveillance remains inconsistently implemented. (2) Objective: To synthesize current molecular dermatology insights relevant to prevention, early detection, and treatment of basal cell carcinoma (BCC) in high-risk survivors, while anchoring the discussion in a detailed case of multiple BCCs after childhood acute lymphoblastic leukemia and HSCT. (3) Methods: Narrative review integrating clinical, dermoscopic, molecular, and translational data from recent high-impact studies; case retained in full. (4) Results: Radiation exposure (especially total body irradiation), prior immunosuppression, and persistent immune dysregulation synergize with ultraviolet mutagenesis to create a “field cancerization” state characterized by Hedgehog-pathway activation (Patched1/Smoothened), impaired Deoxyribonucleic Acid damage response, and stromal remodeling. Dermoscopy, when embedded in routine whole-body examinations, markedly improves accuracy for keratinocyte cancers. Chemoprevention (e.g., nicotinamide) and targeted therapies (hedgehog inhibitors; Programmed Death-1 blockade) represent key translational levers for care innovation. (5) Conclusions: Integrating structured dermatologic surveillance with molecularly informed prevention and therapy should be standard in survivorship pathways for hematopoietic stem cell transplantation/Radiotherapy-exposed patients.

## 1. Introduction

Childhood acute lymphoblastic leukemia (ALL) accounts for approximately 75% of all pediatric leukemia cases, making it the most prevalent malignancy in childhood [1,2,3,4]. Over recent decades, advances in risk-adapted chemotherapy, central nervous system prophylaxis, and hematopoietic stem cell transplantation (HSCT) have driven ALL cure rates beyond 85% in many settings [5,6,7]. Hematopoietic stem cell transplantation (HSCT), often combined with total body irradiation (TBI), remains a cornerstone in high-risk or relapsed disease. However, long-term survivors face increased risks of late effects, including secondary malignant neoplasms (SMNs), immune dysfunction, chronic graft-versus-host disease (GvHD), and therapy-induced comorbidities [8,9,10].

While therapy-related hematologic malignancies such as myelodysplastic syndrome and acute myeloid leukemia have been thoroughly investigated, there is growing recognition of an expanding subset of solid tumors in this population—most notably, cutaneous neoplasms [11,12,13,14,15]. These malignancies, often developing decades after treatment completion, are frequently omitted from structured survivorship surveillance. Among them, basal cell carcinoma (BCC) stands out as the most common skin cancer encountered in childhood ALL survivors, especially those treated with TBI-based HSCT [16,17,18,19]. The pathogenesis of BCC in this setting involves ultraviolet (UV) exposure superimposed on compromised Deoxyribonucleic Acid (DNA) repair capacity, p53 pathway dysfunction, immune dysregulation, and Hedgehog (Hh) signaling activation. Growing evidence indicates that HSCT-related immune impairment and chronic GvHD may create a permissive microenvironment for carcinogenesis, making dermatologic surveillance crucial. The cumulative risk of BCC increases sharply in individuals exposed to ionizing radiation, with TBI estimated to elevate lifetime risk as much as 20-fold compared to the general population [20,21,22,23].

In addition to radiogenic injury, the pathogenesis of cutaneous SMNs is shaped by chronic immune dysregulation and therapeutic immunosuppression. Graft-versus-host disease (GVHD), particularly in its chronic form, creates a microenvironment of persistent inflammation and fibrotic remodeling, further weakening immunologic control over early malignant transformation [8,24,25,26,27]. Immunosuppressive agents, such as calcineurin inhibitors and antimetabolites, have independently been associated with increased skin cancer incidence—either through direct carcinogenicity or by enhancing ultraviolet-mediated mutagenesis [28,29,30,31,32,33]. Despite these converging risk factors, dermatologic follow-up remains sporadic, and skin evaluations are not routinely embedded into survivorship care plans.

As the cohort of childhood leukemia survivors continues to age, the oncologic focus must shift beyond hematologic relapse to encompass a broader spectrum of secondary malignancies. Skin cancer, once considered a rarity in this context, is becoming an increasingly recognized and clinically significant complication—one that requires structured preventive strategies, heightened clinician awareness, and proactive interdisciplinary coordination [34,35,36,37,38].

Although the survival of childhood ALL patients has improved remarkably, the shift in disease burden from relapse to late effects necessitates a comprehensive reevaluation of long-term follow-up frameworks. Current survivorship models often prioritize cardiotoxicity, endocrinopathies, or fertility, while dermatologic malignancies are marginalized despite their growing prevalence. This gap represents not only a clinical oversight but also an emerging field of translational importance, where dermatology and oncology must intersect to optimize patient outcomes.

In addition, cardiovascular disease beyond classic cardiotoxicity has emerged as a major cause of morbidity in long-term HSCT survivors, underscoring the broader spectrum of systemic late effects [39].

Importantly, in the era of precision medicine, cumulative radiation dosage, immuno-modulatory therapies, and genomic susceptibility factors should be integrated into risk stratification algorithms for skin cancer surveillance in this high-risk population.

*Clinical Significance*: The clinical scenario described below is neither isolated nor rare. The convergence of leukemia, HSCT, TBI, prolonged immunosuppression, and GVHD during early life places patients on a uniquely high-risk trajectory for developing cutaneous malignancies. These risks often manifest silently over years, underscoring the need for a paradigm shift in follow-up care. Survivors with such histories should be channeled into targeted dermatologic consultation pathways that include annual full-body skin examinations, dermoscopic monitoring, and patient education in self-surveillance. By integrating dermatologic vigilance into long-term care frameworks, clinicians can facilitate early detection and intervention—mitigating the long-term burden of preventable secondary malignancies in this vulnerable population.

Although survival in childhood ALL now exceeds 85%, the late-effects landscape increasingly features cutaneous SMNs that carry preventable morbidity if detected promptly. Recent cohort analyses of blood or marrow transplant survivors estimate a ~27% 30-year cumulative incidence of any cutaneous malignant neoplasm (≈18% BCC), with risk amplified by TBI, chronic GVHD, and prolonged immunosuppression—highlighting a durable carcinogenic field decades post-therapy [40,41,42,43,44,45].

From a molecular dermatology viewpoint, the convergence of ionizing radiation, ultraviolet (UV) exposure, and immunologic dysregulation activates oncogenic programs (Hedgehog), disables tumor-suppressive checkpoints (e.g., p53), and remodels the dermal niche—conditions that favor clonal expansion of mutated basal cells. This mechanistic framing justifies risk-adapted surveillance and prevention in this population [46,47,48].

In parallel, diagnostic innovation has matured: a large meta-analysis confirms that in-person examination with dermoscopy significantly improves diagnostic accuracy for keratinocyte carcinomas relative to clinical examination alone, supporting routine dermoscopic integration in survivorship clinics [49,50,51,52,53].

Despite abundant survivorship literature, few reports describe multiple, metachronous BCCs developing at a young age after childhood ALL treatment, highlighting the need for increased awareness among dermatologists and oncologists.

The aim of this work is to present a detailed CARE-compliant case of multiple BCCs in a 36-year-old survivor of childhood ALL treated with TBI-based HSCT and to contextualize the findings with a structured review of the current literature.

## 2. Materials and Methods

### 2.1. Study Design

This study consists of a single-patient clinical case report prepared according to the CARE guidelines, accompanied by a structured literature review.

This review was conceived as a clinical–translational synthesis grounded in a single detailed case of multiple BCCs in a long-term survivor of childhood ALL treated with HSCT. The article integrates both clinical and molecular dermatology perspectives, situating the case within the broader scientific literature on SMNs and cutaneous oncology. The design aligns with the standards for narrative reviews in translational medicine and adheres to the PRISMA-ScR (Preferred Reporting Items for Systematic Reviews and Meta-Analyses—Scoping Reviews) conceptual framework for transparency and reproducibility.

Both the CARE Checklist and the PRISMA-ScR checklist were included as materials supporting data processing for reporting transparency.

### 2.2. Ethical Considerations

The patient provided written informed consent for publication of medical data and clinical photographs, with the original Romanian document and English translation submitted to the journal.

The detailed patient case was retrospectively extracted from clinical records at the Dermatology Clinic of the Railways University Hospital, Iași, Romania. All diagnostic, dermoscopic, and histopathologic data were verified by at least two board-certified dermatologists. Written informed consent was obtained from the patient for publication of clinical photographs and anonymized information. Institutional ethical approval was not required as the case represents documentation of routine medical care and aligns with the Declaration of Helsinki’s principles for medical research involving human subjects.

All procedures followed institutional and international ethical standards.

### 2.3. Literature Search Strategy

A structured literature search was conducted in PubMed, Scopus, Web of Science Core Collection (WoS), and the Multidisciplinary Digital Publishing Institute database (to ensure inclusion of dermatology and molecular dermatology journals indexed in WoS). Searches covered January 2010–December 2024 to capture modern HSCT survivorship data and contemporary advances in dermoscopy, molecular diagnostics, and Hedgehog-targeted therapy.

#### 2.3.1. Data Sources and Search Strategy

A comprehensive search of peer-reviewed literature was performed between January 2010 and October 2025 using PubMed, Scopus, Web of Science, and Multidisciplinary Digital Publishing Institute databases. Keywords and Boolean operators included combinations of: basal cell carcinoma, hematopoietic stem cell transplantation, radiation therapy, pediatric leukemia, dermoscopy, field cancerization, Hedgehog signaling, molecular dermatology, and skin cancer prevention. Reference lists of key studies and systematic reviews were also manually screened to identify additional relevant publications. Only English-language articles were considered, and preference was given to studies published in journals indexed in the WoS (Q1–Q2 categories).

A total of 416 records were identified; after screening, 134 articles were included in the final review.

#### 2.3.2. Inclusion and Exclusion Criteria

Inclusion criteria encompassed:

1. Human studies
2. English language
3. Relevance to HSCT survivors or BCC molecular pathogenesis
4. Q1–Q2 dermatology, oncology, hematology, or molecular medicine journals
5. Case reports, cohort studies, mechanistic studies, or reviews
6. Clinical studies, meta-analyses, and experimental reports addressing molecular pathways, diagnostic innovations, or management strategies in BCC or post-transplant skin cancer;
7. Literature concerning survivorship after childhood ALL or HSCT; and
8. Articles discussing palliative care or psychosocial outcomes in pediatric oncology.

Excluded materials comprised:

1. Animal-only studies
2. Conference abstracts
3. Articles without relevance to keratinocyte carcinomas or survivorship
4. Non-peer-reviewed sources
5. Case reports lacking histopathological confirmation
6. Experimental data unrelated to skin malignancy mechanisms.

#### 2.3.3. Analytical Framework

To ensure coherence between clinical and molecular perspectives, evidence was organized across four domains:

1. Epidemiologic and clinical risk factors for cutaneous SMNs;
2. Molecular and genetic pathways implicated in BCC pathogenesis post-radiation or immunosuppression;
3. Diagnostic and dermoscopic innovations, including digital monitoring and AI-based tools; and
4. Therapeutic and preventive strategies within a precision-dermatology framework.

Literature on pediatric palliative care integration, as exemplified by Hizanu Dumitrache et al. (2025) [54], was included to contextualize holistic survivorship care models.

### 2.4. Data Synthesis and Validation

Data extraction focused on identifying recurring mechanistic patterns, diagnostic markers, and clinical management themes. Quantitative findings from cohort and registry studies were summarized descriptively, while mechanistic insights were analyzed qualitatively to delineate emerging molecular targets and clinical translation pathways. The manuscript underwent iterative peer review among co-authors, ensuring multidisciplinary validation across dermatology, hematology, surgery, and molecular biology.

### 2.5. Scientific Integrity and Originality Statement

This manuscript is an original academic synthesis that draws upon verified, cited literature and clinical expertise. All paraphrased materials were rephrased to maintain originality and avoid plagiarism. The authors confirm that no part of the text duplicates previously published content and that intellectual property rights have been respected according to academic publishing standards.

## 3. Case Presentation

### 3.1. Patient History and Background

The patient is a 36-year-old male, Fitzpatrick skin phototype II, diagnosed with high-risk B-cell precursor ALL at age 8. Initial therapy followed a standard Berlin-Frankfurt-Münster (BFM) protocol, including induction, consolidation, CNS prophylaxis, and maintenance. He experienced an early bone marrow relapse and received re-induction chemotherapy followed by allogeneic HSCT. The patient presented for dermatologic evaluation of multiple asymptomatic cutaneous lesions.

This case illustrates the importance of lifelong dermatologic screening in HSCT survivors and outlines molecular, clinical, and therapeutic considerations relevant to their long-term care.

### 3.2. HSCT Details

• Donor: HLA-matched sibling;
• Conditioning regimen: Fractionated TBI (total 12 Gy) + cyclophosphamide;
• Acute GvHD: Grade II (skin + GI), resolved with corticosteroids;
• Chronic GvHD: None;
• Infections: CMV reactivation (treated), no invasive fungal disease;
• TA-TMA: Absent;
• Immunosuppression: cyclosporine for 9 months.

The patient had no family history of BCC, no features of NBCCS, and no history of excessive tanning or occupational UV exposure. The patient had been initially diagnosed with ALL at the age of 14 (in 2003) and underwent first-line multi-agent chemotherapy, achieving complete remission. However, following disease relapse at age 16, he was referred for allogeneic HSCT, which was performed in January 2007 using a fully HLA-identical sibling donor. The pretransplant conditioning regimen included TBI and etoposide, consistent with myeloablative protocols employed during that period.

GVHD prophylaxis was administered via a combination of cyclosporine and methotrexate, following institutional hematologic guidelines. The patient subsequently developed acute GVHD with cutaneous and gastrointestinal involvement, which was effectively managed with systemic corticosteroids. Importantly, no chronic GVHD manifestations were documented in the post-transplant course.

Immunosuppressive therapy was gradually tapered and discontinued following the resolution of acute GVHD, with cyclosporine cessation completed within the standard timeframe advised by the transplant team. By 2014, the patient had entered sustained hematologic remission and was no longer receiving any immunosuppressive agents.

The patient remained clinically stable for over a decade post-HSCT, with no recurrence of leukemia or immune-related complications. The subsequent emergence of multiple BCCs in 2022–2023, occurring approximately seven years after discontinuation of immunosuppression, thus reflects a delayed carcinogenic process likely mediated by the combined long-term effects of ionizing radiation, immune dysregulation, and prior immunosuppressive therapy, rather than active immunocompromise at the time of lesion onset.

### 3.3. Dermatologic Findings

The first BCC appeared at age 34 on the left nasal ala, followed by six additional lesions over the next 18 months on the face, trunk, and scalp. Clinical and dermoscopic features included: arborizing vessels, blue-gray ovoid nests, shiny white structures, superficial scaling (in superficial subtype), ulceration in nodular forms.

**Figure 1:**
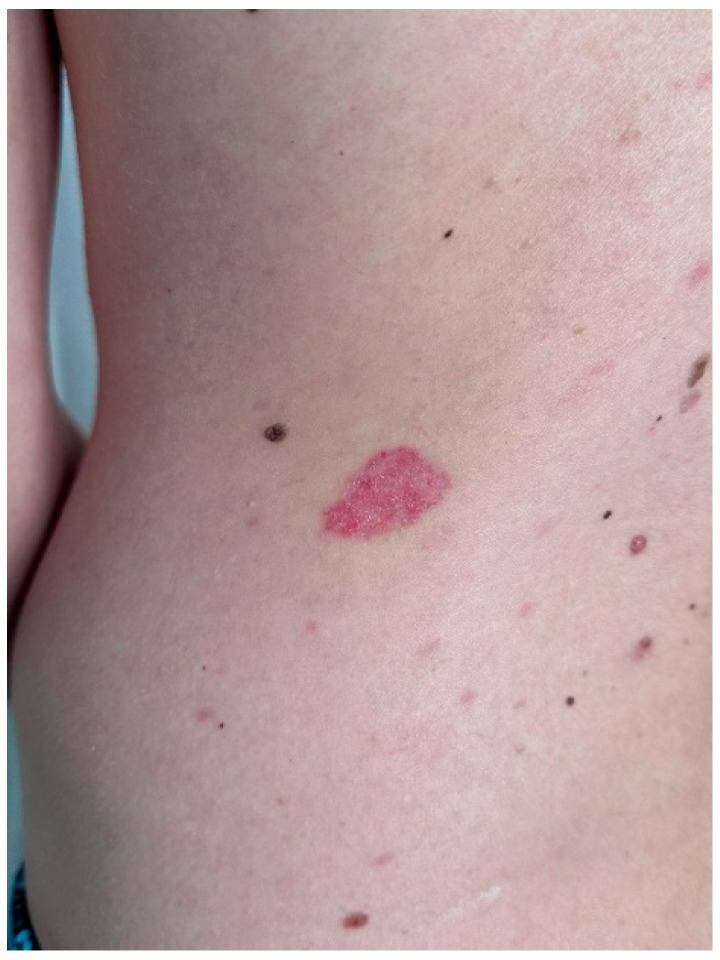
The initial lesion, located in the left lumbar region.

At age 34, the patient noted the development of a nodular lesion in the left lumbar region (Figure 1). Over the subsequent 12 months, nine additional lesions appeared across multiple cutaneous sites, including the back, chest, abdomen, right hip, and presternal region. The lesions varied in size, pigmentation, and surface characteristics.

**Figure 2:**
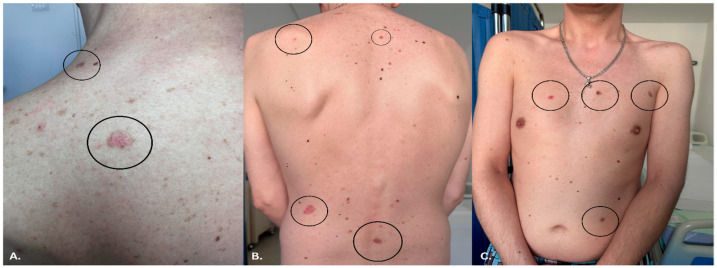
Clinical images showing multiple erythematous-violaceous papules and plaques located on the (**A**) shoulders, (**B**) back, and (**C**) chest. The black circles highlight representative lesions as observed during clinical examination.

**Figure 3:**
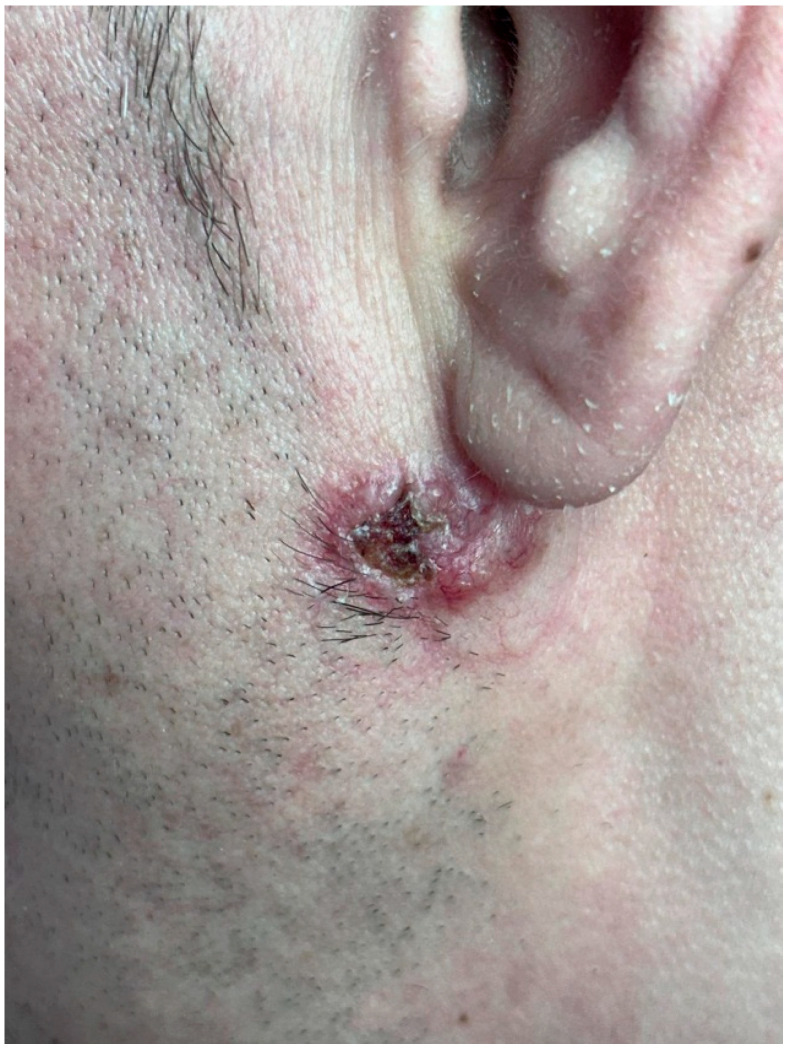
Ulcerated erythemato-violaceous plaque located below the left ear.

Clinical examination revealed ten well-demarcated, elevated papules and plaques, measuring between 0.3 cm and 1.3 cm in diameter (Figure 2A–C). One lesion, located below the left ear, was ulcerated with irregular borders and an erythematous halo (Figure 3). This was the only ulcerated lesion and appeared the most clinically suggestive among all cutaneous findings. The other lesions presented as shiny, pearly papules with varying degrees of pigmentation and surface scaling. No regional lymphadenopathy or systemic symptoms were reported.

The coexistence of lesions with distinct morphologic and dermoscopic signatures in a single patient provides a unique opportunity to observe the natural spectrum of BCC evolution in an immunologically altered, postradiation environment. This underscores the heterogeneity of BCC in transplant survivors compared to sporadic cases, where lesions tend to follow more predictable clinical patterns.

### 3.4. Dermoscopy and Diagnosis

**Figure 4:**
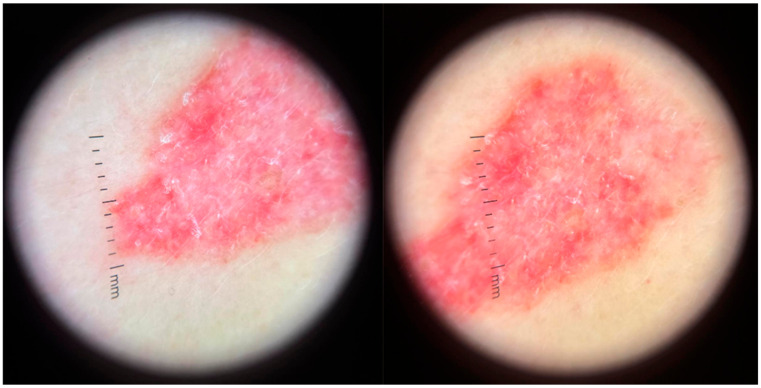
Dermoscopic findings in the lesion located in the left lumbar region (the initial lesion).

**Figure 5:**
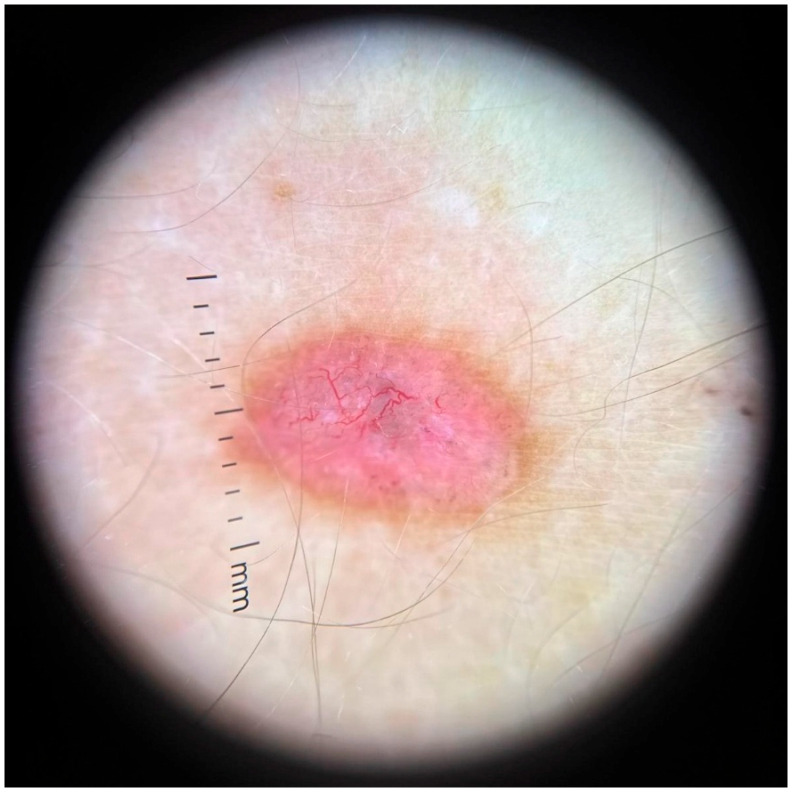
Polymorphous vessels present in one of the lesions located in the lumbar region.

**Figure 6:**
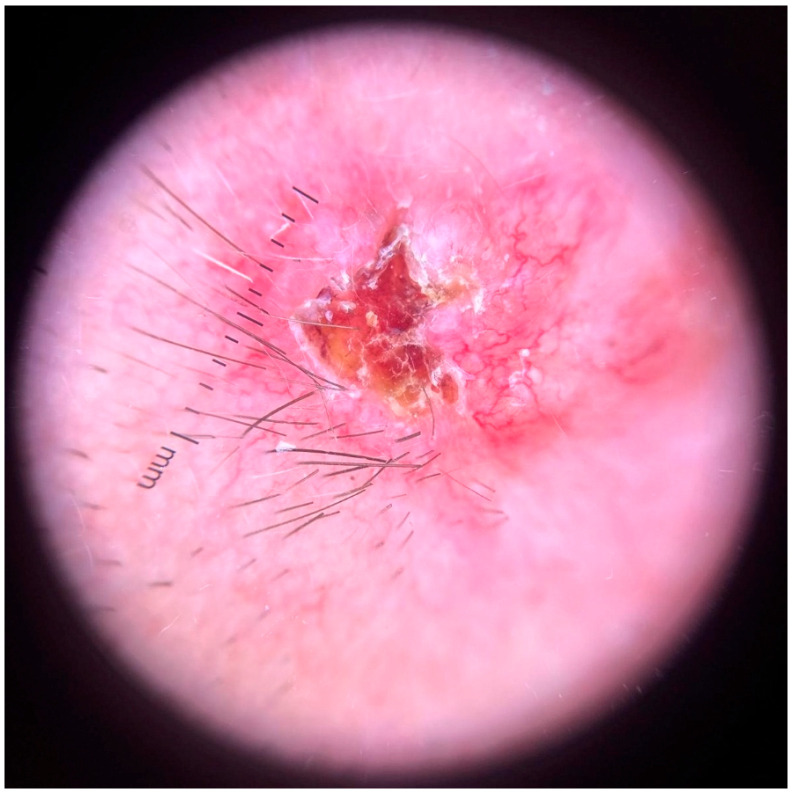
Dermoscopy of the lesion located below the left ear. Chrysalis structures, ulceration and arborizing vessels are present, indicating strong hints for a BCC type tumor.

Dermoscopy revealed a highly polymorphic pattern across the examined lesions, with several key features varying in prominence depending on the lesion’s temporal evolution. The predominant findings included structureless pink areas (Figure 4), often interspersed with polymorphous vascular structures, reflecting the underlying angiogenic activity. Aspects described are structureless pink areas, structureless white areas, discrete squamae—no component is suggestive of classic BCC dermoscopic descriptions. Arborizing vessels, typically associated with BCC, were present in some (Figure 5) but not all lesions (Figure 4). These were accompanied by shiny white streaks, also known as chrysalis structures, which correspond to dermal fibrosis and were more pronounced in certain lesions (Figure 6).

Additionally, ovoid nests were identified in several lesions, appearing as well defined, dark brown to black structures suggestive of aggregations of basal cell nests within the epidermis. Structureless brown areas were also observed, varying in intensity and distribution, potentially indicative of melanin dispersion. Some lesions displayed white regression areas, characterized by a loss of pigmentation, likely reflecting immune-mediated tumor regression.

**Figure 7:**
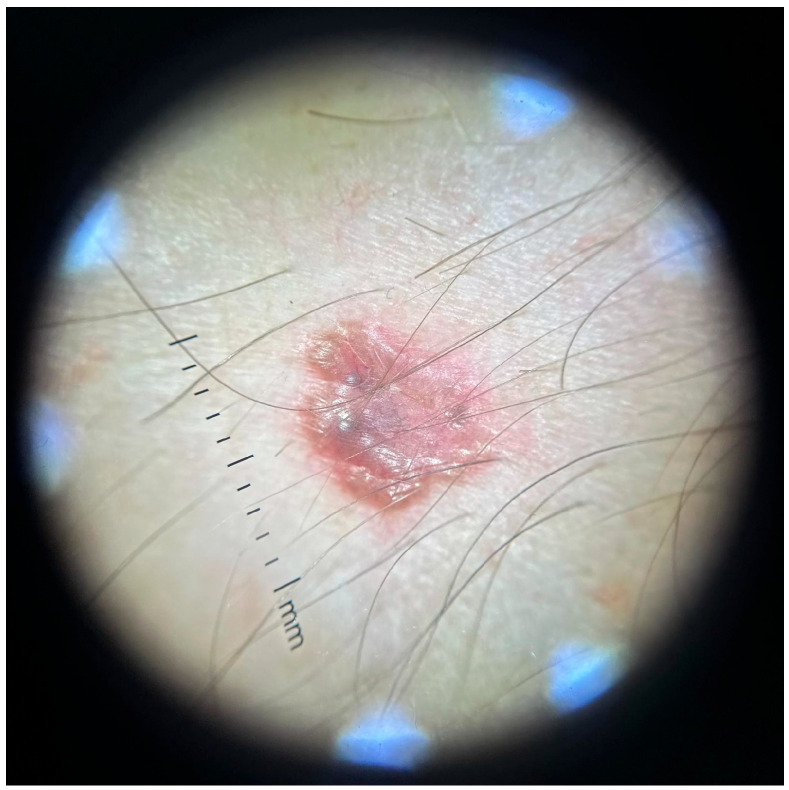
Pearly crown visible in non-polarized light dermoscopy of a lesion located in the presternal region.

A distinct pearly crown at the periphery was noted in some lesions, manifesting as a translucent, light-reflecting rim, a feature often seen in nodular BCC (Figure 7).

Notably, the lesions developed in a sequential manner rather than simultaneously, with newer lesions exhibiting more vascularized and erythematous patterns, whereas older lesions displayed increased fibrosis and regression features. The patient was able to indicate the approximate order of lesion appearance, allowing for an assessment of the dermoscopic progression over time. Lesions that emerged in close temporal succession exhibited similar dermoscopic characteristics, whereas those that developed over a longer interval demonstrated more pronounced differences, positioning them on a continuous spectrum of dermoscopic evolution.

### 3.5. Histopathology

Punch and excisional biopsies confirmed nodular and superficial BCC subtypes, showing basaloid cell clusters with peripheral palisading, mucinous stroma, focal ulceration, and stromal retraction.

### 3.6. Management and Follow-Up

Lesions were treated with standard surgical excision and curettage. No Hh inhibitors were required (Table 1).

## 4. Discussion and Literature Review

### 4.1. Risk of BCC After Childhood ALL and HSCT

Childhood cancer survivors carry increased SMN risk; HSCT and TBI amplify this tendency, particularly for BCC. Studies demonstrate a 10–30-fold increased BCC incidence compared to age-matched controls. Younger age at radiation and fair skin phototypes contribute significantly.

This case highlights an underrecognized but clinically significant manifestation of long-term survivorship in patients treated for childhood acute lymphoblastic leukemia—namely the development of multiple basal cell carcinomas decades after hematopoietic stem cell transplantation. While the oncologic community has long been attuned to therapy-related hematologic malignancies, there is growing awareness of secondary solid tumors, with cutaneous malignancies emerging as a particularly relevant subset. The cumulative risk of non-melanoma skin cancers is notably increased among survivors who underwent total body irradiation as part of their conditioning regimen, with some studies reporting incidence rates up to 20-fold higher than those in non-irradiated controls. In a cohort of 3880 Blood or Marrow Transplantation (BMT) survivors, the 30-year cumulative incidence of any cutaneous malignant neoplasm was ~27.4% (18% for BCC), with cGVHD, immunosuppression, and age at transplant as significant risk factors [8,16,40,55,56,57,58]. The Boull et al. report gives cumulative incidence estimates and treatment/treatment-related risk factors in childhood cancer survivors (<21 yrs) [59]. Consistent with Thorsness et al., low-risk histologic subtypes predominate, recurrence is rare, and lesions are most often located in prior radiotherapy (Radiation Therapy, RT) fields [60].

**Figure 8:**
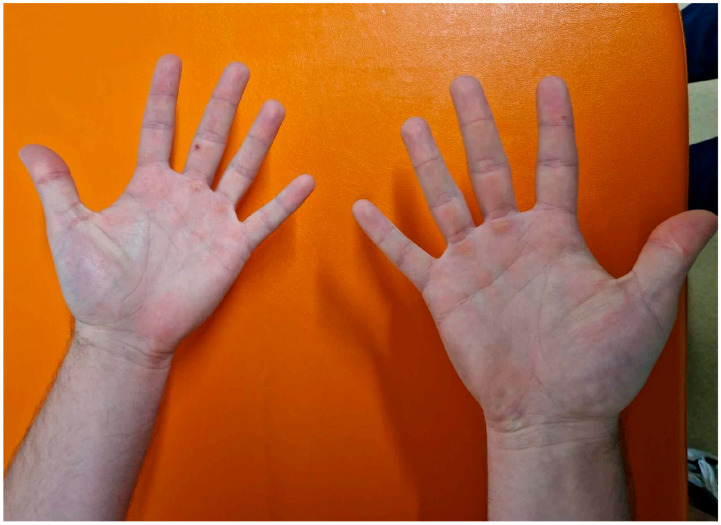
Clinical image of the patient’s palms showing smooth, uninterrupted epidermal surface with no evidence of palmar pits. The absence of these hallmark features supports exclusion of Nevoid Basal Cell Carcinoma Syndrome (Gorlin Syndrome) as part of the differential diagnosis.

Given the unusually high number of BCCs identified in this patient, we carefully considered the possibility of Nevoid Basal Cell Carcinoma Syndrome (NBCCS), also known as Gorlin-Goltz syndrome. According to established diagnostic criteria, NBCCS requires the presence of at least two major criteria or one major and two minor criteria. In our case, the only fulfilled major criterion was the presence of multiple BCCs. The patient lacked palmar or plantar pits (documented clinically and supplemented with palmar photographs, Figure 8), had no histologically confirmed odontogenic keratocysts, and no imaging evidence suggestive of lamellar calcification of the falx cerebri or rib anomalies. Furthermore, no first-degree relatives were affected by NBCCS or related cutaneous syndromes.

With respect to the minor criteria, the patient exhibited no signs of macrocephaly, congenital facial malformations (such as frontal bossing, hypertelorism, cleft lip or palate), skeletal deformities, radiographic anomalies, or neurologic sequelae such as medulloblastoma. These features were assessed through comprehensive physical examination, review of historical imaging, and multidisciplinary input (including hematology and dermatology). To preserve patient anonymity, facial images were withheld, but clinical documentation confirms absence of dysmorphic features or ocular abnormalities. As such, this constellation of findings fails to satisfy the diagnostic threshold for NBCCS and strongly supports an acquired, non-syndromic etiology for the multiple BCCs in this case.

Importantly, this patient developed ten distinct BCCs over a one-year period, all exhibiting clinical and dermoscopic heterogeneity. This polymorphism may complicate early diagnosis, especially in the absence of routine dermatologic surveillance. Notably, the lesions did not arise simultaneously but appeared in a sequential fashion, suggesting a progressive and persistent carcinogenic field effect likely driven by prior TBI exposure, cumulative UV radiation, and immunologic dysfunction. This underscores the long latency and multifactorial nature of cutaneous SMNs in post-transplant populations [24,60,61,62].

The role of graft-versus-host disease and its management further amplifies risk. Chronic inflammation and long-term immunosuppression impair cutaneous immune surveillance, diminishing the skin’s ability to detect and clear emerging tumor clones. While the patient in this case did not exhibit chronic GVHD, his prior use of systemic corticosteroids and cyclosporine during a vulnerable developmental period may have contributed to durable immune dysregulation, further enhancing susceptibility to skin neoplasia [24,28,63,64,65,66,67]. Highlights field cancerization, risk of multiple NMSCs in immunosuppressed transplant recipients; parallels with HSCT patients, especially immunosuppression and surveillance [68,69,70].

Despite these well-established risk factors, current post-HSCT surveillance strategies rarely prioritize dermatologic assessment, particularly in adult survivors of pediatric malignancies. This gap may lead to underdiagnosis or delayed intervention, allowing lesions to reach more advanced stages before detection. The case presented here supports calls for the integration of structured skin cancer screening into survivorship protocols, particularly for patients with histories of TBI and immunosuppression [11,45,71,72,73,74]. Full-body skin examination, annual dermoscopy, and digital monitoring should become standard practice in this population, coupled with patient education on UV protection and self-examination. The meta-analysis of 100 studies revealed that experienced dermatologists employing dermoscopy achieve sensitivity and specificity for keratinocytic cancers substantially higher than with clinical examination alone [49,51,61,75,76,77,78,79,80].

Moreover, the high lesion burden observed in this patient raises important questions about chemoprevention and early intervention strategies. While surgical excision remains the gold standard for BCC management, future studies should explore the role of topical agents, photodynamic therapy, or systemic HHIs in patients with multiple or recurrent lesions, particularly when surgical morbidity is high.

From a research standpoint, this case reinforces the need for large-scale longitudinal studies investigating the long-term dermatologic outcomes of pediatric cancer survivors. Further work is needed to quantify the incidence, latency, and risk factors for cutaneous SMNs across various treatment regimens. There is also an unmet need to validate dermoscopic criteria specific to radiation- and immunosuppression-associated BCCs, which may differ in morphology from sporadic cases.

This case exemplifies the concept of a “field cancerization effect” in irradiated and immunologically compromised skin, where sequential, multifocal carcinogenesis occurs over time. The phenomenon suggests that ongoing mutagenic stress and impaired DNA repair mechanisms continue to drive tumorigenesis decades after the initial lesion.

Beyond clinical implications, this report advances an innovative perspective: dermoscopic polymorphism in post-HSCT patients should be recognized as a potential diagnostic hallmark. Developing dermoscopic algorithms specifically tailored to radiation- and immunosuppression-related BCCs could improve early recognition in this population.

Recent transcriptional profiling has identified distinct molecular signatures in high-risk aggressive BCCs, including dysregulation of pathways relevant to Hedgehog signaling and immune checkpoint activation [81,82,83,84,85,86,87]. Furthermore, the translational potential of chemopreventive strategies—such as systemic retinoids, nicotinamide supplementation, or immunomodulatory interventions—deserves exploration through multicenter clinical trials. Such preventive approaches may substantially reduce surgical burden and morbidity, especially in patients with multiple synchronous or metachronous lesions [88,89,90,91].

This case operationalizes the concept of radiation- and immunosuppression-primed field cancerization, in which sequential BCCs arise over years with dermoscopic heterogeneity. Population-level data from transplant and childhood cancer survivor cohorts now quantify this risk and justify proactive, life-long skin surveillance [40,45,59,92,93,94,95].

Methodologically, the strongest evidence for improving early detection is the routine use of dermoscopy by trained clinicians, which should be embedded into survivorship guidelines and electronic care pathways. On the preventive side, nicotinamide represents a practical chemopreventive tool to discuss with high-risk survivors, while systemic Hedgehog inhibition and, where appropriate, Programmed Death-1 (PD-1) therapy extend options for anatomically challenging or recurrent disease [49,61,96,97,98,99,100,101,102].

### 4.2. Molecular Pathogenesis in HSCT Survivorship

BCC pathogenesis in this context reflects the interaction of UV-induced DNA damage with therapy-induced genomic vulnerability.

Key mechanisms include: Hedgehog pathway activation (Patched1 loss, Smoothened mutations), p53 mutations from radiation exposure, impaired DNA repair capacity after TBI, altered immune surveillance due to HSCT and prior immunosuppression

#### Molecular Dermatology Perspective: Pathways, Biomarkers, and the “Field”

Ionizing radiation (RT/TBI) induces complex DNA lesions (double-strand breaks) and persistent oxidative stress; together with UV signatures (e.g., C > T transitions), this accelerates driver events in Patched1 (PTCH1)/Smoothened (SMO) (Hedgehog), TP53, and other genomic loci relevant to BCC. The post-transplant immune milieu further weakens immunosurveillance, fostering clonal persistence and recurrence potential. Clinically, this manifests as multifocal, metachronous BCCs in irradiated fields—precisely the pattern observed in our patient—supporting a durable field cancerization model. Large survivor cohorts corroborate these clinical patterns, including elevated keratinocyte carcinoma burden after childhood cancer, particularly with prior RT [59,103,104,105].

Translational biomarkers under study include genomic profiling (PTCH1/SMO mutations), circulating tumor DNA for difficult sites, and stromal/immune signatures that may predict responsiveness to hedgehog inhibition or immunotherapy. While not yet standard for routine BCC care, these tools are increasingly relevant for complex, multi-lesional disease in survivorship settings [106,107,108,109].

### 4.3. Dermoscopy in High-Risk Populations

Dermoscopy is essential for early detection. HSCT survivors may show: multiple lesion morphologies, subtle early BCC indicators, rapid evolution of superficial lesions.

In high-risk survivors, dermoscopic polymorphism may reflect lesion age, microenvironmental fibrosis, and variable Hedgehog pathway activation; documenting sequential evolution with digital dermoscopy supports earlier biopsy thresholds and staged management. Systematic review data reinforce that dermoscopy meaningfully raises sensitivity/specificity for keratinocyte cancers compared with clinical inspection alone, especially in expert hands, making it indispensable for HSCT/RT cohorts [49,110].

### 4.4. Therapeutic Options

A new figure summarizing BCC treatments has been added, covering: surgery, Mohs micrographic surgery, Hedgehog inhibitors (vismodegib, sonidegib), immune checkpoint inhibitors (cemiplimab), photodynamic therapy, field therapy (imiquimod, 5-FU).

The patient has been referred to a plastic surgery clinic to initiate the surgical removal of all identified skin tumors. Given the multiplicity and varied topography of the lesions, a staged excision approach has been recommended. Priority will be given to the most clinically significant lesions—those that are larger, more infiltrative, ulcerated, or located in functionally or cosmetically sensitive areas, such as the preauricular lesion beneath the left ear. Subsequent interventions will target lesions that are less prominent, located in covered anatomical regions, or demonstrate features of early-stage basal cell carcinoma. The decision-making process in the surgical management will integrate clinical, dermoscopic, and histopathological factors to optimize both oncologic control and esthetic outcomes.

Equally critical in the comprehensive management of this patient is ongoing hematologic supervision. Periodic assessments by the hematology team remain essential to ensure sustained remission of acute lymphoblastic leukemia and to monitor for late complications of hematopoietic stem cell transplantation, including secondary malignant neoplasms, bone marrow dysfunction, and chronic GVHD manifestations.

The integration of pediatric palliative care (PPC) into oncologic and post-transplant follow-up is increasingly recognized as a critical dimension of comprehensive care. A retrospective analysis of 2022–2023 Romanian data demonstrated that early, multidisciplinary PPC interventions improve quality of life, psychosocial adaptation, and long-term family outcomes for children facing life-limiting conditions. Within the context of molecular dermatology and long-term cancer survivorship, PPC should be reframed not merely as end-of-life support but as a continuum of care encompassing symptom management, psychological counseling, and educational guidance for both patients and families. In survivors of childhood leukemia and HSCT, chronic dermatologic sequelae—such as radiation-induced skin fibrosis, multiple carcinomas, and psychosocial distress—can persist well into adulthood. Integrating PPC principles into dermatologic survivorship care enables earlier identification of suffering (physical or emotional), improved adherence to preventive strategies, and enhanced coordination between oncology, dermatology, and psychosocial services. This approach aligns with a precision-medicine philosophy in which clinical innovation coexists with compassionate, human-centered care, ensuring that advances in molecular dermatology are translated into meaningful, patient-oriented outcomes [54].

### 4.5. Dermatologic Surveillance in Survivorship

Dermatologic follow-up at regular intervals is indispensable. Annual full-body skin exams are recommended; high-risk survivors may require biannual surveillance, sun protection counseling, and photographic monitoring. Shared-care models between dermatology and survivorship clinics improve outcomes.

Given the patient’s demonstrated propensity for developing multiple basal cell carcinomas within a short timeframe, lifelong surveillance is warranted. This should include routine total body skin examinations with dermoscopy, documentation of evolving or new lesions through clinical photography, and a low threshold for biopsy of atypical findings. Emphasis should be placed on patient education, encouraging self-examination and prompt reporting of new or changing skin lesions. Adjunctive tools such as digital dermoscopy and sequential monitoring may enhance early detection in this high-risk population.

Beyond staged surgical excision, a precision–prevention framework is warranted:

1. Photoprotection and UV avoidance;
2. Chemoprevention—randomized Phase III evidence supports oral nicotinamide (500 mg twice daily) in patients with prior NMSC to reduce new lesions during active treatment;
3. Risk-adapted intervals for full-body dermoscopy;
4. Consideration of topical field therapies (5-FU, imiquimod, daylight-PDT) for actinic field control;
5. Early multidisciplinary evaluation for unresectable/morbid lesions, including hedgehog inhibitors or PD-1 blockade where indicated [101,102,111,112,113,114].

For locally advanced or periorbital tumors, hedgehog inhibitors (vismodegib, sonidegib) achieve meaningful responses but require proactive toxicity management and adherence strategies; newer reviews outline long-term optimization models [115,116,117,118].

#### 4.5.1. Innovative Diagnostics: Digital Dermoscopy, AI, and Longitudinal Monitoring

Embedding dermoscopy into survivorship clinics is supported by high-quality evidence; the incremental value is greatest when paired with longitudinal digital imaging and standardized descriptors. Meta-analytic data show that dermatologist performance improves substantially with dermoscopy versus unaided exam, with implications for triage, earlier detection, and reduced unnecessary biopsies [49,80,119,120].

Pragmatically, HSCT/RT-exposed survivors benefit from: baseline total-body photography; lesion mapping with serial dermoscopy; risk-tiered recall (6–12 months) escalated by UV history, radiation fields, and immunosuppression history; and teledermoscopy adjuncts for interim changes.

#### 4.5.2. Translational Therapeutics and Precision Prevention

Hedgehog pathway inhibitors (HHIs: vismodegib, sonidegib) remain first-line systemic options for advanced BCC; emerging strategies emphasize intermittent dosing, toxicity mitigation, and combination approaches to enhance durability while preserving quality of life [100,114,121,122,123].

Chemoprevention: The ONTRAC Phase III RCT supports nicotinamide 500 mg BID to reduce new NMSC events during treatment in high-risk populations—an attractive, low-cost option for irradiated survivors, with the caveat that benefits wane after discontinuation [101,124,125,126].

Immuno-oncology: For HHI-refractory disease or contraindications, PD-1 blockade has activity in advanced BCC; patient selection may be refined by tumor mutational burden and immune microenvironment features in future studies [127,128,129,130].

Field therapy and surgery: Algorithmic integration of field therapies (e.g., daylight-PDT) with staged excisions can reduce cumulative surgical morbidity in multi-lesional patients, while maintaining oncologic control [131,132,133,134].

## 5. Conclusions and Future Perspectives

This case highlights cutaneous malignancies—particularly basal cell carcinoma (BCC)—as a significant yet still underrecognized late complication in long-term survivors of childhood acute lymphoblastic leukemia (ALL) treated with hematopoietic stem cell transplantation (HSCT), radiotherapy, and prolonged immunosuppression. The development of multiple, clinically heterogeneous BCCs decades after oncologic therapy illustrates the persistent carcinogenic vulnerability of this population and underscores the concept of long-lasting field cancerization in previously irradiated and immunologically altered skin.

Lifelong dermatologic surveillance should therefore be regarded as an essential component of survivorship care rather than an optional adjunct. The systematic integration of structured skin cancer screening—including regular full-body examinations, dermoscopy-assisted assessment, and patient education for self-surveillance—can facilitate earlier diagnosis, reduce cumulative surgical morbidity, and improve long-term quality of life. These measures are particularly critical for survivors exposed to total body irradiation or chronic immunosuppression, who remain at elevated risk well into adulthood.

The findings of this report support the need for an interdisciplinary survivorship model in which dermatologists, oncologists, hematologists, and transplant specialists collaborate to develop standardized, risk-adapted guidelines for skin cancer prevention and early detection. Advances in molecular dermatology, including targeted inhibition of the Hedgehog pathway, immunotherapeutic strategies, and evidence-based chemoprevention, offer opportunities to complement surgical management and reduce disease burden in selected high-risk patients.

Looking forward, future research should prioritize longitudinal cohort studies to better define cumulative risk trajectories, identify molecular and genetic susceptibility markers—such as PTCH1 or SMO variants—and validate precision–prevention approaches. The integration of digital surveillance tools, including artificial intelligence–assisted dermoscopy and teledermatology platforms, may further enhance early detection while expanding access to specialized care, particularly in resource-limited settings. As the population of childhood cancer survivors continues to grow, proactive, molecularly informed dermatologic care will be pivotal in preventing avoidable morbidity from otherwise treatable skin malignancies.

## Figures and Tables

**Table 1:** Chronological timeline of diagnostic procedures and treatment approaches related to cutaneous malignancies.

Year/Period	Age	Clinical Event	Diagnostic Procedures	Histopathologic Findings	Therapeutic Interventions
2007	Childhood	Treatment for acute lymphoblastic leukemia	Hematologic evaluation; imaging	—	Allogeneic HSCT following conditioning with total body irradiation (12 Gy) and cyclophosphamide
2007–2008	Childhood	Acute graft-versus-host disease	Clinical assessment (skin, gastrointestinal tract)	—	Systemic immunosuppression (cyclosporine, corticosteroids); aGvHD graded according to modified Glucksberg criteria (Grade II)
2014	Young adult	Long-term remission	Routine hematologic follow-up	—	Discontinuation of immunosuppressive therapy
2022	34	First cutaneous lesion detected (left lumbar region)	Full-body clinical examination; dermoscopy; skin biopsy	Basal cell carcinoma, nodular subtype	Complete surgical excision
2022–2023	34–35	Sequential development of multiple skin lesions (*n* = 10)	Repeated full-body examinations; serial dermoscopic assessments	Multiple basal cell carcinomas (nodular and superficial subtypes)	Staged surgical excisions; curettage where appropriate
2023	35	Multidisciplinary management	Dermatologic and plastic surgery evaluation	Confirmatory histopathology of excised lesions	Surgical management; systemic therapies (Hedgehog pathway inhibitors) not indicated
Ongoing	—	Survivorship follow-up	Regular dermatologic surveillance; dermoscopy-assisted monitoring	—	Lifelong follow-up; patient education; self-skin examination; hematologic survivorship care

## Data Availability

No new data were created or analyzed in this study. Data sharing is not applicable to this article due to patient privacy and ethical considerations.

## Abbreviations

The following abbreviations are used in this manuscript:

5-FU	5-fluorouracil
AI	Artificial Intelligence
ALL	Acute Lymphoblastic Leukemia
BCC	Basal Cell Carcinoma
BFM	Berlin-Frankfurt-Münster
BMT	Blood or Marrow Transplantation
DNA	Deoxyribonucleic Acid
GVHD	Graft-Versus-Host Disease
HHI	Hedgehog pathway Inhibitor
HSCT	Hematopoietic Stem Cell Transplantation
NBCCS	Nevoid Basal Cell Carcinoma Syndrome
NMSC	Non-Melanoma Skin Cancer
ONTRAC	Oral Nicotinamide to Reduce Actinic Cancer
PD-1	Programmed Death-1
PDT	Photodynamic Therapy
PPC	Pediatric Palliative Care
PRISMA-ScR	Preferred Reporting Items for Systematic Reviews and Meta-Analyses—Scoping Reviews
PTCH1	Protein patched homolog 1
RT	Radiation Therapy (Radiotherapy)
SMNs	Secondary Malignant Neoplasms
SMO	Smoothened = a 7-transmembrane protein essential to the activation of Gli transcription factors (Gli) by hedgehog morphogens
TBI	Total Body Irradiation
TP53	A gene that codes for the tumor protein p53
UV	Ultraviolet
